# A kinase inhibitor screen identifies a dual cdc7/CDK9 inhibitor to sensitise triple-negative breast cancer to EGFR-targeted therapy

**DOI:** 10.1186/s13058-019-1161-9

**Published:** 2019-07-01

**Authors:** Ronan P. McLaughlin, Jichao He, Vera E. van der Noord, Jevin Redel, John A. Foekens, John W. M. Martens, Marcel Smid, Yinghui Zhang, Bob van de Water

**Affiliations:** 1 Department of Drug Discovery and Safety, Leiden Academic Centre for Drug Research, Leiden University, 2300 RA Leiden, The Netherlands; 2 Department of Medical Oncology and Cancer Genomics Netherlands, Erasmus MC Cancer Institute, Erasmus University Medical Center, Rotterdam, The Netherlands

**Keywords:** CDK9/Cdc7 inhibition, EGFR-targeted therapy, Drug resistance, Triple-negative breast cancer

## Abstract

**Background:**

The effective treatment of triple-negative breast cancer (TNBC) remains a profound clinical challenge. Despite frequent epidermal growth factor receptor (EGFR) overexpression and reliance on downstream signalling pathways in TNBC, resistance to EGFR-tyrosine kinase inhibitors (TKIs) remains endemic. Therefore, the identification of targeted agents, which synergise with current therapeutic options, is paramount.

**Methods:**

Compound-based, high-throughput, proliferation screening was used to profile the response of TNBC cell lines to EGFR-TKIs, western blotting and siRNA transfection being used to examine the effect of inhibitors on EGFR-mediated signal transduction and cellular dependence on such pathways, respectively. A kinase inhibitor combination screen was used to identify compounds that synergised with EGFR-TKIs in TNBC, utilising sulphorhodamine B (SRB) assay as read-out for proliferation. The impact of drug combinations on cell cycle arrest, apoptosis and signal transduction was assessed using flow cytometry, automated live-cell imaging and western blotting, respectively. RNA sequencing was employed to unravel transcriptomic changes elicited by this synergistic combination and to permit identification of the signalling networks most sensitive to co-inhibition.

**Results:**

We demonstrate that a dual cdc7/CDK9 inhibitor, PHA-767491, synergises with multiple EGFR-TKIs (lapatinib, erlotinib and gefitinib) to overcome resistance to EGFR-targeted therapy in various TNBC cell lines. Combined inhibition of EGFR and cdc7/CDK9 resulted in reduced cell proliferation, accompanied by induction of apoptosis, G2-M cell cycle arrest, inhibition of DNA replication and abrogation of CDK9-mediated transcriptional elongation, in contrast to mono-inhibition. Moreover, high expression of cdc7 and RNA polymerase II Subunit A (POLR2A), the direct target of CDK9, is significantly correlated with poor metastasis-free survival in a cohort of breast cancer patients. RNA sequencing revealed marked downregulation of pathways governing proliferation, transcription and cell survival in TNBC cells treated with the combination of an EGFR-TKI and a dual cdc7/CDK9 inhibitor. A number of genes enriched in these downregulated pathways are associated with poor metastasis-free survival in TNBC.

**Conclusions:**

Our results highlight that dual inhibition of cdc7 and CDK9 by PHA-767491 is a potential strategy for targeting TNBC resistant to EGFR-TKIs.

**Electronic supplementary material:**

The online version of this article (10.1186/s13058-019-1161-9) contains supplementary material, which is available to authorized users.

## Background

Triple-negative breast cancer (TNBC) is a notoriously aggressive, heterogeneous disease defined as lacking expression of oestrogen receptor (ER) and/or progesterone receptor (PR) as well as amplification of human epidermal growth factor receptor 2 (HER2), respectively [1]. Although TNBC only constitutes approximately 15–20% of breast cancer cases, it is disproportionately responsible for breast cancer-associated deaths and carries a dismal prognosis, compared with hormone receptor-positive (HR+) breast cancers [2–4]. For patients with HR+ breast cancer, endocrine therapy targeting ER is available in the form of aromatase inhibitors and selective oestrogen receptor modulators (e.g. tamoxifen) and other antagonists [1]. Contrastingly, no effective targeted therapy which exploits the molecular properties of tumour cells exists for TNBC patients; clinical trials of targeted agents in TNBC have been disappointing [5, 6]. Consequently, aggressive chemotherapy, radiotherapy and surgery remain the mainstay treatments [7]. Furthermore, patients who develop resistance to treatment, or who do not respond to treatment whatsoever, follow an aggressive clinical course characterised by metastasis and a higher 5-year mortality post-diagnosis [8]. Further complicating the treatment of TNBC is the degree of genetic heterogeneity observed in this disease. By analysing the gene expression profiles of TNBC cases, Lehmann et al. sub-classified TNBC into six different molecular subtypes: mesenchymal (M), mesenchymal stem-like (MSL), luminal androgen receptor-positive (LAR), immunomodulatory (IM), basal-like 1 (BL1) and basal-like 2 (BL2) [9]. Most importantly, these subtypes exhibit dissimilar drug-sensitivity profiles, resulting in varied clinical responses [9, 10]. The nature of TNBC clearly necessitates a more tailored approach to treatment, one which exploits the unique oncogenic addictions present. For chemotherapy-resistant TNBC patients, the development of targeted therapeutics which synergise with current treatment options to overcome resistance is therefore paramount.

Epidermal growth factor receptor (EGFR; also known as ERBB1/HER1) is often expressed at higher levels in triple-negative tumours than in HR+ tumours [11], though expression levels vary, with up to ~ 80% of TNBC cases reported as being EGFR+ [12]. EGFR amplification has also been reported to occur in a substantial proportion of TNBC cases [13–15] with EGFR overexpression associated with a much poorer prognosis in general [8]. Furthermore, EGF signalling is highly enriched in the basal and mesenchymal TNBC subtypes [9]. EGFR therefore represents a bona fide drug target in triple-negative tumours. Various EGFR inhibitors have been developed, most notably anti-EGFR monoclonal antibodies (e.g. cetuximab) and EGFR-tyrosine kinase inhibitors (EGFR-TKIs) (e.g. erlotinib, gefitinib and lapatinib) [16]. Despite these efforts, EGFR-TKI single treatment has performed poorly in clinical trials for TNBC patients with advanced or metastatic breast cancer, despite clear inhibition of EGFR [17, 18] suggesting bypass inhibition of EGFR-related signalling in TNBC tumours [19, 20]. Nonetheless, EGFR-TKIs have shown more promising results as combination therapies in TNBC [21], perhaps indicating that monotherapeutic inhibition of EGFR is insufficient to shut down the myriad signalling pathways responsible for promoting aberrant proliferation and survival.

Here we demonstrate that multiple EGFR-TKIs synergise with the dual cdc7/CDK9 inhibitor PHA-767491 in a panel of TNBC cell lines resistant to EGFR-TKIs. This combination inhibited cell proliferation, induced apoptosis and G2-M arrest and downregulated components critical to cell cycle progression, DNA replication and transcription, thereby reversing resistance to EGFR-TKIs. Therefore, targeting deficiencies in regulation of the cell cycle and DNA replication in conjunction with transcriptional addiction downstream of growth factor pathways may constitute a powerful therapeutic opportunity for this difficult-to-treat breast cancer subtype.

## Methods

### Cell culture

All cell lines were maintained in RPMI-1640 medium (Gibco, ThermoFisher Scientific, Breda, The Netherlands) supplemented with 10% FBS (Thermo Fisher Scientific; 10270106) and 25 IU/ml penicillin and 25 μg/ml streptomycin (ThermoFisher Scientific; 15070-063). Cells were cultured in a humidified incubator at 37 °C, 5% CO_2_. Cell lines were provided by Erasmus MC Rotterdam and tested monthly for mycoplasma using PCR.

### Antibodies and kinase inhibitors

The primary antibodies against pEGFR (Y1173, #4407), pERK1/2 (T202/Y204, #9101), ERK1/2 (#4695), pAKT (S473, #9271), AKT (#9272), MCM2 (#3619), pRNA-II (S2/5; #4735), RNA-II (#2629), and p-pRb (S780, # 9307) were commercially supplied from Cell Signaling TECHNOLOGY®, EGFR (sc-03), pRb (sc-102), CDK4 (sc-601), Cyclin D1 (sc-20,044) from Santa Cruz BIOTECHNOLOGY®, cdc7 (ab10535) and pMCM2 (S40/41; ab70371) from Abcam® and Tubulin (T-9026) from Sigma®. Secondary antibodies included Cy5-conjugated anti-mouse and horseradish peroxidase (HRP) anti-mouse or anti-rabbit (Jackson ImmunoResearch). Individual kinase inhibitors lapatinib (S2111), gefitinib (S1025), erlotinib (S7786) and PHA-767491 (S2742), plus the previously described 273-kinase inhibitor library (L1200), were purchased from Selleckchem® (Munich, Germany) and dissolved in DMSO solution at 10 mM [22]. TAK-931 and BAY-1143572 were purchased from MedChemExpress (Sollentuna, Sweden).

### Kinase inhibitor treatment and drug combination screen

Cells were seeded into 96-well plates at the appropriate densities (Additional file 1: Table S1). The following day, cells were treated with individual kinase inhibitors in dose range as indicated. Vehicle DMSO (1:1000) was used as control. For the kinase inhibitor library screen, cells were screened in duplicate against the kinase inhibitor library containing 273 kinase inhibitors at concentration of 1 μM alone, or the 1 μM library inhibitors in combination with lapatinib at 3.16 μM, since this concentration effectively inhibited EGFR phosphorylation in all cell lines tested and since studies have shown that levels of lapatinib in patient tumours vary between 1 and 12 μM depending on dosing schedule [23]. After 4-day treatment, proliferation was evaluated by sulphorhodamine B (SRB) colorimetric assay [24] and analysed by % of control cell growth = (mean sample OD − mean 0-day OD)/(mean control OD − mean 0-day OD) × 100. To assess synergistic interaction of combined drugs, combination index (CI) analysis [25, 26] was performed, using the formula ‘CI = (D)1/(Dx)1 + (D)2/(Dx)2’. (D)1 and (D)2 are respective combination doses of two compounds that yield an effect of 50% of proliferation inhibition, with (Dx)1 and (Dx)2 being the corresponding single doses for either compound that results in the same effect, which is by definition the IC50 of each compound. CI values less than 1 (CI < 1), equal to 1 (CI = 1) or greater than 1 (CI > 1), indicate synergy, additivity or antagonism, respectively.

### Western blotting

Cells were seeded in 6-well plates at the appropriate density. For stimulation/starvation assays, medium was refreshed with serum-free medium (SFM) the following day and cells were starved overnight. Thereafter, cells were pre-treated with drug solutions for 4 h, then stimulated with 100 ng/ml EGF (Sigma; E9644) for 5 min in SFM. For time-course exposures to drugs, cells were treated with drug solutions prepared in complete medium. Cell lysates were harvested at the indicated time points in RIPA lysis buffer with 1:100 Protease Inhibitor Cocktail (Sigma; P8340). Cellular proteins were denatured in sample buffer containing 10% β-mercaptoethanol, loaded with 30 μg/lane into 7.5% polyacrylamide gels, resolved using SDS-PAGE and subsequently transferred to PVDF membranes (Merck Chemicals; IPVH00010) overnight. PVDF membranes were then blocked with 5% BSA-TBST (Tris-buffered saline 0.05% Tween-20) and subsequently incubated at 4 °C overnight with appropriate primary antibodies. The following day, membranes were incubated for 1 h with HRP- or Cy5-conjugated secondary antibodies and chemiluminescence or fluorescence was detected using the Las4000 (GE Healthcare).

### Annexin-V staining

Cells were seeded overnight in 96-well μCLEAR plates (Corning) at appropriate densities, then treated with drug solutions at indicated concentrations. At 24, 48 or 72 h post-treatment, cells were stained with Hoechst 33258 (1:10,000) and Annexin-V (1:1000) for 45 min at 37 °C, 5% CO_2_ before being imaged using BD Pathway 855 Microscope (BD Biosciences). Annexin-V staining was quantified using Cell Profiler software.

### Cell cycle flow cytometry analysis

Cells were seeded in 6-well plates at the appropriate density. Twenty-four or 48 h post-treatment, all cells were harvested, re-suspended in ice-cold 200 μl 1 mM EDTA-PBS and 800 μl 100% ethanol, and stored at − 20 °C before being centrifuged at 1000 rpm at 4 °C. Cells were then re-suspended in 1 ml PBS and rehydrated for 15 min. After being spun at 1000 rpm for 5 min at room temperature, the pellet was re-suspended in 250 μl 3 mM DAPI (Sigma, 10236276001) staining buffer (100 μM Tris pH 7.4, 150 mM NaCl, 1 mM CaCl_2_, 0.5 mM MgCl_2_), incubated for 15 min at room temperature in the dark, followed by filtration through 70-μm EASYstrainer filters and analysed using FACS Conto II (*BD Biosciences).* Data were analysed using FlowJo V10.

### siRNA transfection

Cells were seeded in 96-well plates at the appropriate density. For each siRNA transfection, 50 nM siGENOME siRNAs (Dharmacon) were transfected into cells per 96-well using INTERFERin transfection reagent (Polyplus; 409-50). The following day, the medium was refreshed. Forty-eight hours post-transfection, cells were either lysed for western blot to confirm knockdown or treated with drugs for the appropriate duration as described, then fixed for SRB proliferation assay.

### Clinical evaluation of candidate target genes

The clinical relevance of cdc7, POLR2A and CDK9 was evaluated using in-house gene expression and metastasis-free survival data of 123 lymph node-negative, non-(neo) adjuvantly treated, oestrogen receptor-negative (ER-neg) primary breast cancer patients. The composition of this cohort is described in Additional file 2: Table S2. The clinical relevance of synergy-related candidate genes was evaluated using the previously described in-house as well as publicly available gene expression and MFS data of lymph node-negative, non-(neo) adjuvantly treated primary breast cancer patients, leading to a cohort of 142 triple-negative patients. Data were gathered from Gene Expression Omnibus (http://www.ncbi.nlm.nih.gov/geo/) entries GSE2034, GSE5327, GSE2990, GE7390 and GSE11121, with all data available on Affymetrix U133A chip. Raw.cel files were processed using fRMA parameters (median polish) [27] after which batch effects were corrected using ComBat [28].

### Transcriptome RNA sequencing and pathway integration analysis

Cells were seeded overnight in 6-well plates and treated in triplicate for 6 h with individual or combined kinase inhibitors at indicated concentrations, or vehicle. RNA was isolated with RNeasy Plus Mini Kit as described by the manufacturer (QIAGEN, Cat. 74136). Transcriptome RNA sequencing (RNA-Seq) was performed using Illumina high-throughput RNA sequencing. DNA libraries were prepared from the samples with the TruSeq Stranded mRNA Library Prep Kit. The DNA libraries were sequenced according to the Illumina TruSeq v3 protocol on an Illumina HiSeq2500 sequencer. Paired-end reads of 100bp in length were generated. Alignment was performed against the human GRCh38 reference genome using the STAR aligner (version 2.4.2a). Marking duplicates, sorting and indexing were performed using sambamba. Gene expression was quantified using the FeatureCounts software (version 1.4.6) based on the ENSEMBL gene annotation for GRCH38 (release 84). RNA-Seq data was normalised by TMM using EdgeR’s normalisation factor [29], followed by quantile normalisation and presented in Log2 fold change (Log2 FC) scales. Genes with significant down- or upregulation (Log2 FC ≥ |0.5|) under indicated conditions were analysed by web-based functional analysis tool Ingenuity pathway Analysis (IPA) to visualise and annotate their biological functions and pathways.

### Statistical analyses

All statistical analyses, where appropriate, were performed in GraphPad Prism software version 7.0. One-way ANOVA multiple comparison test with Tukey’s post hoc test was applied with *p* values less than 0.05 considered as statistically significant.

## Results

### TNBC cells are resistant to EGFR-TKIs

**Fig. 1:**
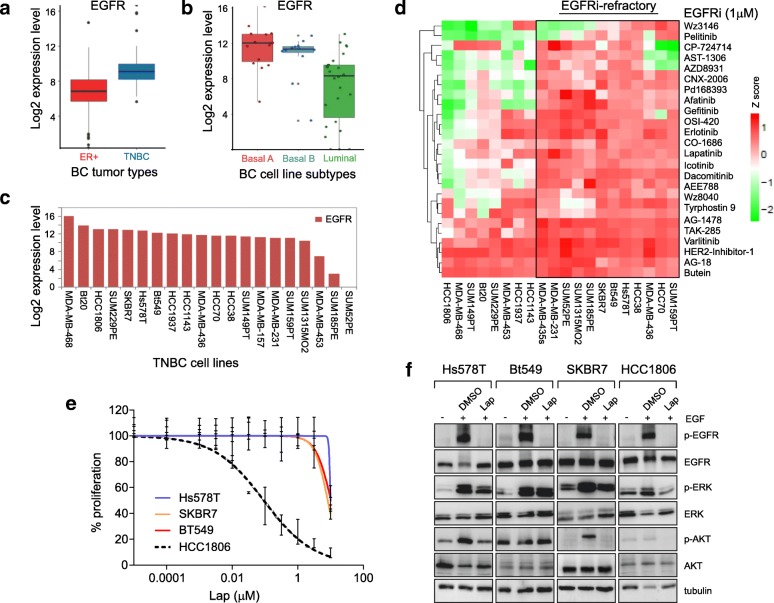
Resistance of TNBC cells to EGFR-TKIs. **a** RNA-Seq-based log_2_ EGFR expression levels in oestrogen receptor-positive (ER+) breast cancer (991 cases) and TNBC tumours (116 cases) derived from 1107 cases in The Cancer Genome Atlas (TCGA) database. Graphs show data distribution, the mean and the lower and upper quartiles. Error bars represent standard deviation. **b** RNA-Seq-based log_2_ EGFR expression in basal-like and luminal-like BC cell line subtypes (total 50 BC cell lines, in-house). Graphs show data distribution, the mean and the lower and upper quartiles. Error bars represent standard deviation **c** EGFR expression in 20 TNBC cell lines. **d** Resistance of TNBC cells to EGFR inhibitors (EGFRi). Cells were treated with 1 μM inhibitor for 4 days. Proliferation was assessed using sulphorhodamine B (SRB) assay. *Z*-score was represented by normalising raw values to those of DMSO control. **e** Dose response of EGFRi-resistant TNBC cell lines (Hs578T, BT549 and SKBR7) and an EGFRi-sensitive cell line (HCC1806) to lapatinib. Percentage of proliferation was normalised to DMSO control. Data shown as mean ± standard deviation for two independent experiments performed in triplicate. **f** EGFR pathway functionality in TNBC cell lines Hs578T, BT549 and SKBR7 and HCC1806. Cells were starved for 24 h in serum-free medium, treated with drug solutions prepared in serum-free medium for 4 h then stimulated with 100 ng/ml EGF for 5 min

EGFR is expressed at higher levels in TNBC tumours compared to ER-positive BC tumours (Fig. 1a); also in human basal A and basal B TNBC cell lines, there is a higher EGFR expression than in human luminal cell lines (Fig. 1b). Therefore, we sought to systematically elucidate the response of TNBC to a broad range of different EGFR kinase inhibitors. A panel of TNBC cell lines with varying EGFR expression (Fig. 1c was screened against 24 EGFR-TKIs (Fig. 1d). Twelve cell lines (> 57%) could be classified as refractory to almost all 24 EGFR-TKIs; only HCC1806 was highly sensitive to most EGFR-TKIs, the remainder having variable sensitivity to EGFR-TKIs (Fig. 1d). Next, three TNBC cell lines highly resistant to EGFR-TKIs, Hs578T, BT549 and SKBR7, and one sensitive cell line, HCC1806, were selected for further evaluation. Hs578T, BT549 and SKBR7 cells were resistant to lapatinib-mediated growth inhibition up to and including concentrations of 3.16 μM, but superior concentrations (10 μM) impeded cellular proliferation (Fig. 1e). Concordantly, lapatinib failed to significantly induce apoptosis in these cell lines at 3.16 μM (Additional file 3: Figure S1a). In contrast, HCC1806 cells displayed enhanced growth inhibition in response to lapatinib (IC50 ~ 100 nM; Fig. 1e) with significantly increased Annexin-V apoptotic signal (Additional file 3: Figure S1a). Regardless of their response to lapatinib, all these cell lines maintained functional EGFR-mediated signal transduction, with prominent phosphorylation of EGFR (Y1173) and downstream components AKT (S473) and ERK1/2 (T202/Y204) in response to EGF stimulation (Fig. 1f), indicating that resistance was not due to the absence of a functionally intact EGFR pathway. In response to lapatinib, EGFR phosphorylation was completely inhibited in all cell lines (Fig. 1f). However, EGF-induced ERK activation persisted in all lapatinib-resistant cell lines, with AKT phosphorylation also unaffected in Hs578T and BT549 cells. These data suggest that these resistant cells are capable of bypassing EGFR kinase inhibition through the activation of downstream pro-proliferative pathways. Despite the lack of impact of EGFR-TKIs on TNBC proliferation, siRNA-mediated silencing of EGFR and downstream components, including ERK2 and FRAP1 (mTOR), led to a significant reduction in cell proliferation, supporting the notion that TNBC cells depend to a certain extent on EGFR-mediated signalling for their proliferation (Additional file 3: Figure S1b).

### Kinase inhibitor combination screening identifies a dual cdc7/CDK9 inhibitor PHA-767491 which synergises with lapatinib in TNBC

**Fig. 2:**
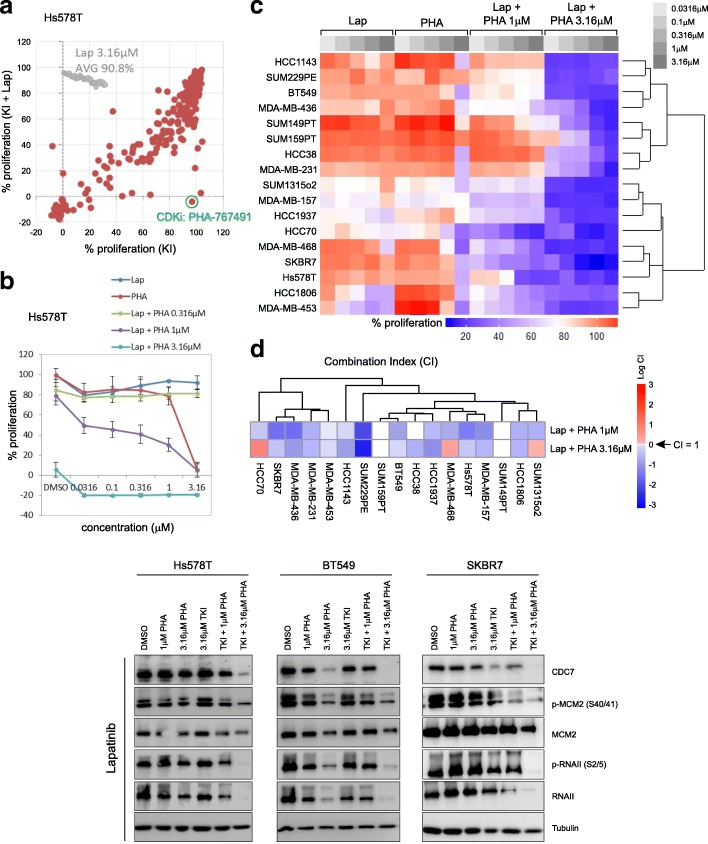
Kinase inhibitor combination screening identifies compounds which synergise with EGFR-TKIs in TNBC. **a** EGFRi lapatinib (Lap) and kinase inhibitor (KI) combination screen in EGFRi-resistant Hs578T cells. Cells (8000/well) were treated with 1 μM of 273 individual KIs alone, or in combination with lapatinib at 3.16 μM, for 4 days. The dual cdc7/CDK9 inhibitor PHA-767491, one of the KIs which most strongly synergised with lapatinib, is singled out, as indicated. **b** Dose response curves for Hs578T cells. Cells were treated with a dose range of PHA-767491 or lapatinib (0.0316–3.16 μM) or lapatinib combined with 0.316, 1 or 3.16 μM PHA-767491. Data shown as mean ± standard deviation for two independent experiments. **c** Validation screen of lapatinib/PHA-767491 synergistic effect in a panel of 17 TNBC cell lines, under indicated combinations. Cells were treated with dose ranges of lapatinib or PHA-767491 (0.0316–3.16 μM) or the indicated combinations. **d** Combination indices (CI) for lapatinib (3.16 μM) combined with either 1 μM or 3.16 μM PHA-767491. Log CI is shown. **e** Initiation of DNA replication and productive transcriptional elongation in Hs578T, BT549 and SKBR7 cells treated with lapatinib and/or PHA-767491 as indicated, for 48 h. TKI refers to lapatinib at 3.16 μM

Next, we sought to identify compounds which synergise with lapatinib by performing a combinatorial kinase inhibitor screen in Hs578T cells treated with 273 kinase inhibitors at 1 μM with or without 3.16 μM lapatinib. Most notably, amongst a number of compounds which augmented the response of TNBC cells to EGFR inhibition, including JAK3 inhibitor WHI-P154, AKT/PDPK1 inhibitor PHT-427, PIKfyve inhibitor YM201636, pan-AKT inhibitor GSK690693, JAK2 inhibitor TG101348 and Aurora A/B/C kinase inhibitor PHA-680632, the dual cdc7/CDK9 inhibitor PHA-767491 inhibited proliferation by > 90% compared to control or either monotherapy (Fig. 2a). Subsequent dose response experiments revealed that combinations of 1–3.16 μM PHA-767491 with 0.0316–3.16 μM lapatinib greatly enhanced inhibition of Hs578T growth compared to either monotherapy (Fig. 2b), indicative of synergy, whereas the synergistic effects of the other candidate compounds did not pass validation. To confirm this interaction was not cell line-specific, 17 TNBC cell lines were screened with a dose range of lapatinib or PHA-767491 (0.0316–3.16 μM) or with a dose range of lapatinib in combination with either 1 μM or 3.16 μM PHA-767491. These TNBC cell lines were generally resistant to both monotherapies at doses equal to or less than 1 μM, but lapatinib and PHA-767491 co-treatment strongly inhibited proliferation (Fig. 2c). Combining lapatinib (3.16 μM) with PHA-767491 at either 1 μM or 3.16 μM led to strong synergistic responses in the vast majority of TNBC cell lines (with some additive responses also evident), yielding CI values well below 1 (Fig. 2d), thus confirming the synergistic nature of this interaction.

### Dual pharmacological inhibition of EGFR and cdc7/CDK9 suppresses components of the transcriptional machinery and DNA-replicative program

Having confirmed the synergistic effect of PHA-767491 and lapatinib on TNBC proliferation, we elucidated the impact of co-treatment on EGFR-, cdc7- and CDK9-mediated signalling transduction. Forty-eight hours post-exposure, lapatinib (3.16 μM) in combination with PHA-767491 (1 μM) reduced levels of cdc7, accompanied by decreased phosphorylation of its downstream target MCM2 (Ser40/41), a critical component of DNA helicase, suggesting an inhibitory effect on initiation of DNA replication in Hs578T, BT549 and SKBR7 cells (Fig. 2e). This co-treatment also moderately decreased CDK9-mediated phosphorylation of Ser2/5 residues in the C-terminal domain of RNA Polymerase II (RNAII), implying impairment of CDK9- and/or CDK7-mediated productive transcriptional elongation (Fig. 2e). Additionally, co-treatment with PHA-767491 at 3.16 μM not only led to complete abolition of cdc7 levels but also depletion of total MCM2 and RNAII and their associated phosphorylated forms, perhaps indicative of stalled RNAII-mediated transcriptional elongation (Fig. 2e). These data suggest that co-treatment of TNBC cells with lapatinib and the dual cdc7/CDK9 inhibitor PHA-767491 blocks components essential for initiation of DNA replication and RNA polymerase II-mediated transcription.

### Selective EGFR-TKIs erlotinib and gefitinib also synergise with PHA-767491 in TNBC

**Fig. 3:**
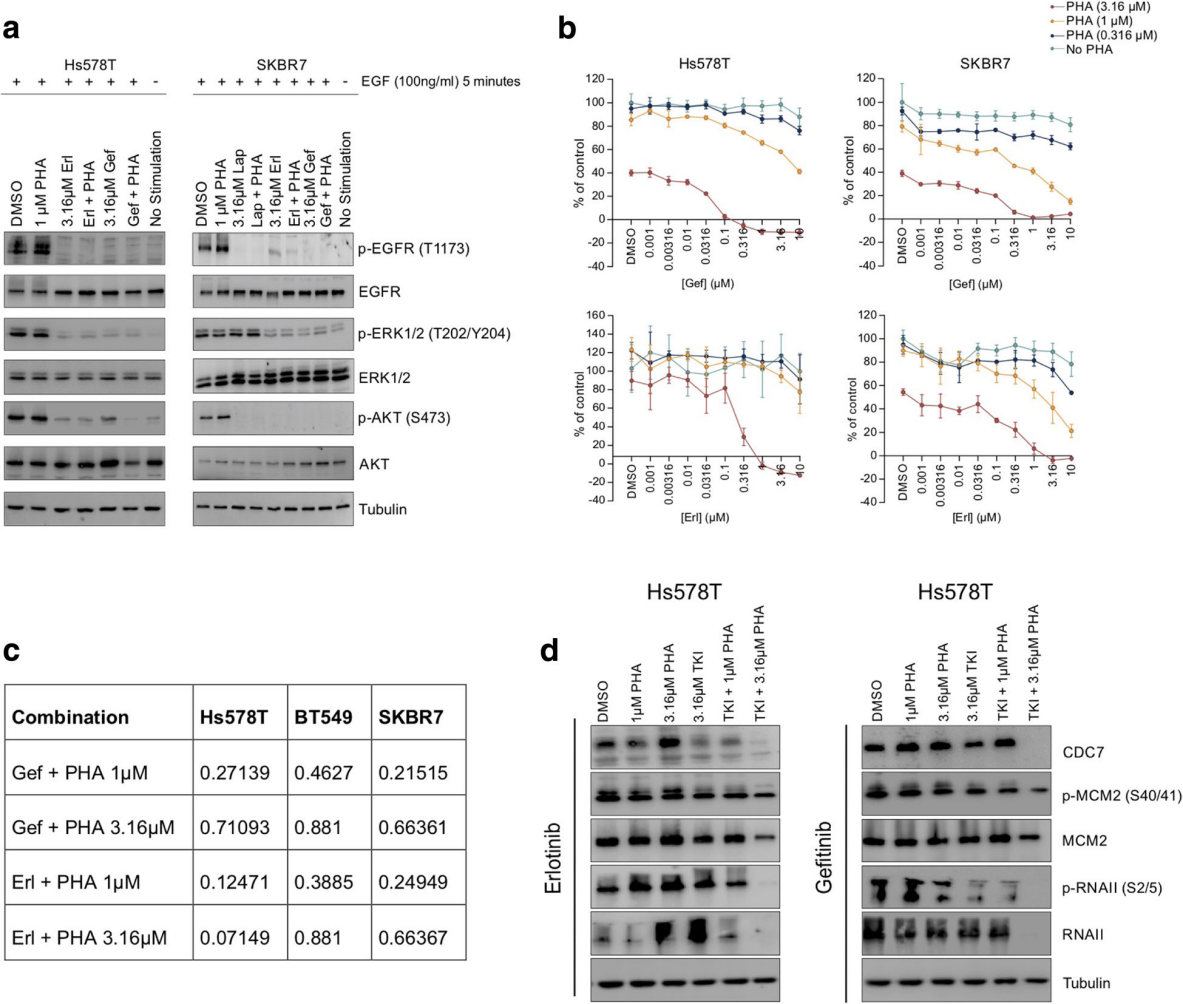
Selective EGFR-TKIs erlotinib and gefitinib also synergise with PHA-767491 in TNBC. **a** Inhibitory impact of erlotinib and gefitinib on EGFR-mediated signal transduction in Hs578T and SKBR7 cells. **b** Dose response of Hs578T and SKBR7 cells to gefitinib or erlotinib (0.0316–3.16 μM) combined with 0.316, 1 or 3.16 μM PHA-767491. Data shown as mean ± standard deviation. Each graph shows one representative of two independent experiments performed in triplicate. Combination indices (CI) of gefitinib (3.16 μM) or erlotinib (3.16 μM) with PHA-767491 (1 μM or 3.16 μM) in Hs578T, BT549 and SKBR7 cells. **d** Initiation of DNA replication and productive transcriptional elongation in Hs578T cells treated with erlotinib or gefitinib and/or PHA-767491 as indicated, for 48 h. TKI refers to either erlotinib or gefitinib at 3.16 μM

To verify whether synergy between lapatinib and PHA-767491 represents a general EGFR-TKI-related phenomenon, TNBC cells were treated with other selective EGFR-TKIs, erlotinib and gefitinib [30], in combination with PHA-767491. At a concentration of 3.16 μM, erlotinib and gefitinib inhibited EGF-mediated activation of EGFR phosphorylation and downstream phosphorylation of ERK1/2 and AKT in Hs578T and SKBR7 cells (Fig. 3a), perhaps reflective of their increased potency compared to lapatinib in eliciting EGFR inhibition. However, proliferation of all cell lines tested was refractory to erlotinib and gefitinib monotherapy up to 3.16 μM (Fig. 3b). Strikingly, at concentrations of 0.1 μM or above, erlotinib and gefitinib strongly synergised with 1 μM and 3.16 μM PHA-767491, almost completely inhibiting the proliferation of Hs578T and SKBR7 cells (Fig. 3b, c). The effect of combining erlotinib or gefitinib with PHA-767491 on DNA replication and transcription-related signal transduction largely resembled that of lapatinib combined with PHA-767491 (Fig. 3d). Only combinations of erlotinib or gefitinib and PHA-767491 were capable of decreasing cdc7 levels and consequently phosphorylation of MCM2 (Ser40/41). Similarly, inhibition of CDK9-mediated RNA-II (S2/S5) phosphorylation was most prominent after co-treatment. To validate the hypothesis that EGFR inhibition is critical for the synergistic interaction between EGFR-TKIs and PHA-767491, an EGFR-negative triple-negative cell line, SUM185PE (Additional file 4: Figure S2a), was treated with this combination. Neither AKT nor ERK1/2 was activated in response to stimulation with EGF, confirming EGF-mediated signalling is redundant in SUM185PE (Additional file 4: Figure S2b). As expected, lapatinib and PHA-767491 did not synergise in EGFR-negative SUM185PE cells (Additional file 4: Figure S2c).

### Dual treatment of TNBC cells with EGFR-TKIs and PHA-767491 induces G2/M cell cycle arrest and apoptosis

**Fig. 4:**
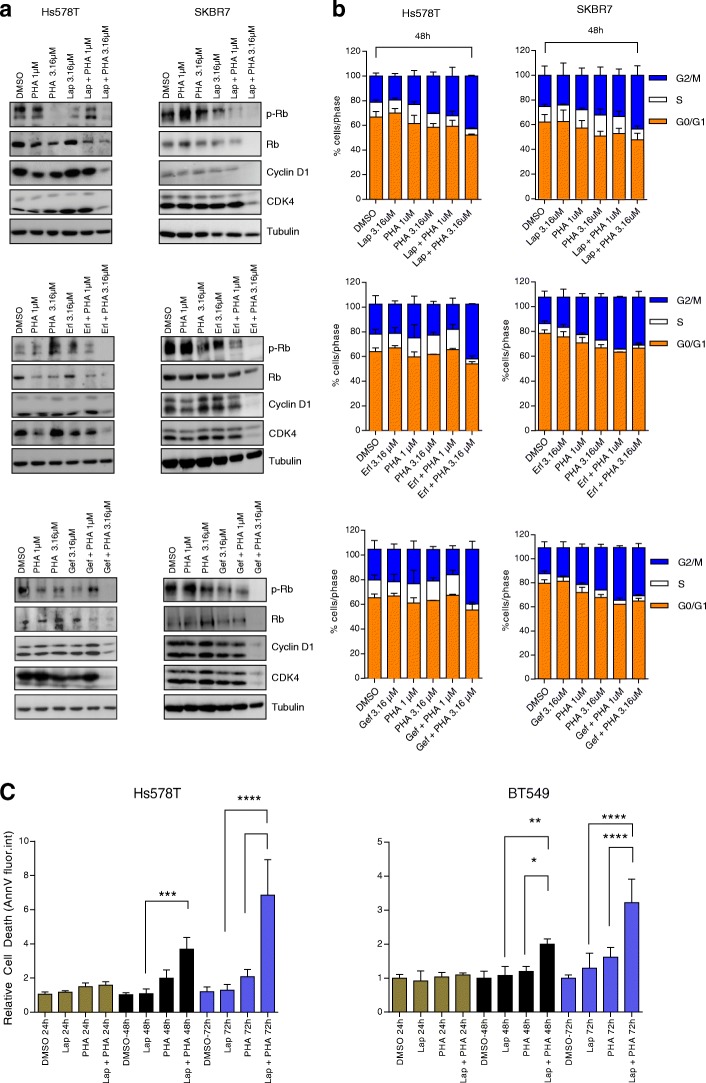
EGFR-TKIs and PHA-767491 synergise to induce G2/M cell cycle arrest and apoptosis. **a** Inhibition of cell cycle components by co-treatment with EGFR-TKIs (lapatinib, erlotinib or gefitinib) and PHA-767491. Hs578T and SKBR7 cells were treated with EGFR-TKI alone (3.16 μM) or combined with PHA-767491 (1 μM or 3.16 μM) for 48 h as indicated. **b** Cell cycle distribution of Hs578T and SKBR7 cells after 48-h treatment with EGFR-TKIs (3.16 μM) alone or combined with PHA-767491 (1 μM or 3.16 μM), as indicated. Data shown as mean of two independent experiments ± standard deviation. **c** Induction of apoptosis by EGFR-TKI and PHA-767491 co-treatment. Hs578T and BT549 cells were treated with lapatinib (3.16 μM), PHA-767491 (3.16 μM), alone or combined, as indicated, for 24 h, 48 h or 72 h, respectively, and then stained with Annexin-V and Hoechst, followed by imaging and image quantification. Data shown as mean ± standard deviation of two independent experiments. Relative cell death was quantified by normalising the intensity of Annexin-V signal to that of DMSO control. One-way ANOVA ****P ≤ 0.0001, ***P ≤ 0.001, **P ≤ 0.01, *P ≤ 0.05

Whilst PHA-767491 at 3.16 μM inhibits cdc7 and phosphorylation of MCM2 and RNA polymerase II, parallel inhibition of EGFR was necessary for eliciting a similar effect on cell cycle components. Decreased levels of CDK4, Cyclin D1 and phosphorylated pRb were observed 48 h post-exposure to co-treatment (Fig. 4a), suggesting concomitant inhibition of EGFR, cdc7 and CDK9 obstructs cell cycle progression in TNBC cells. Flow cytometric analysis confirmed that G2-M cell cycle arrest occurred only when lapatinib, erlotinib or gefitinib was combined with 3.16 μM PHA-767491 in Hs578T and SKBR7 cells (Fig. 4b; Additional file 5: Figure S3a). Accordingly, Annexin-V staining indicated that monotherapies failed to induce appreciable levels of apoptosis in lapatinib-resistant TNBC cells, whilst combination treatments enhanced apoptosis (Fig. 4c; Additional file 5: Figure S3b). Subsequently, RNAi-mediated silencing of cdc7 and CDK9 was used to dissect their respective contributions to the observed synergy. siCdc7 and siCDK9 in combination with lapatinib significantly reduced SKBR7 proliferation compared to knockdown alone, though no sensitisation was seen in Hs578T or BT549 cells (Additional file 6: Figure S4a-b). siCdc7 or siCDK9 reduced cdc7 or CDK9 expression as well as reduced levels of MCM2 (S40/41) phosphorylation (Additional file 6: Figure S4c). However, neither cdc7 nor CDK9 knockdown alone in combination with lapatinib fully recapitulated the impact of combined lapatinib and PHA-767491 treatment on signal transduction. Whilst MCM2 activation was inhibited by cdc7 knockdown, minimal inhibition of RNAII (S2/5) phosphorylation was observed after CDK9 knockdown and CDK4 levels remained constant for both cdc7- and CDK9-depleted cells following lapatinib treatment (Additional file 6: Figure S4c). Nevertheless, also knockdown of the main off-targets of PHA-767491 did not synergise with lapatinib (Additional file 6: Figure S4d). As the biological effect of knockdown (e.g. depletion of protein) and inhibition (inhibition of activity of protein) differs, we additionally tested the highly selective cdc7 inhibitor, TAK-931, and CDK9 inhibitor, BAY-1143572 together with lapatinib. Whilst low concentrations of these compounds (0.316 μM and 0.1 μM resp.) had limited effect on proliferation, nor upon addition of lapatinib (3.16 μM), simultaneous treatment with these cdc7, CDK9 and EGFR inhibitors strongly affected TNBC cell proliferation (Additional file 6: Figure S4e). Nevertheless, these effects were less pronounced than the combination of PHA-767491 with lapatinib, suggesting that possibly the broad spectrum kinase inhibitor activity of PHA-767491 contributes to the strong synergistic effect.

### Expression of cdc7 and RNAII (POLR2A) is linked to poor prognosis in breast cancer

**Fig. 5:**
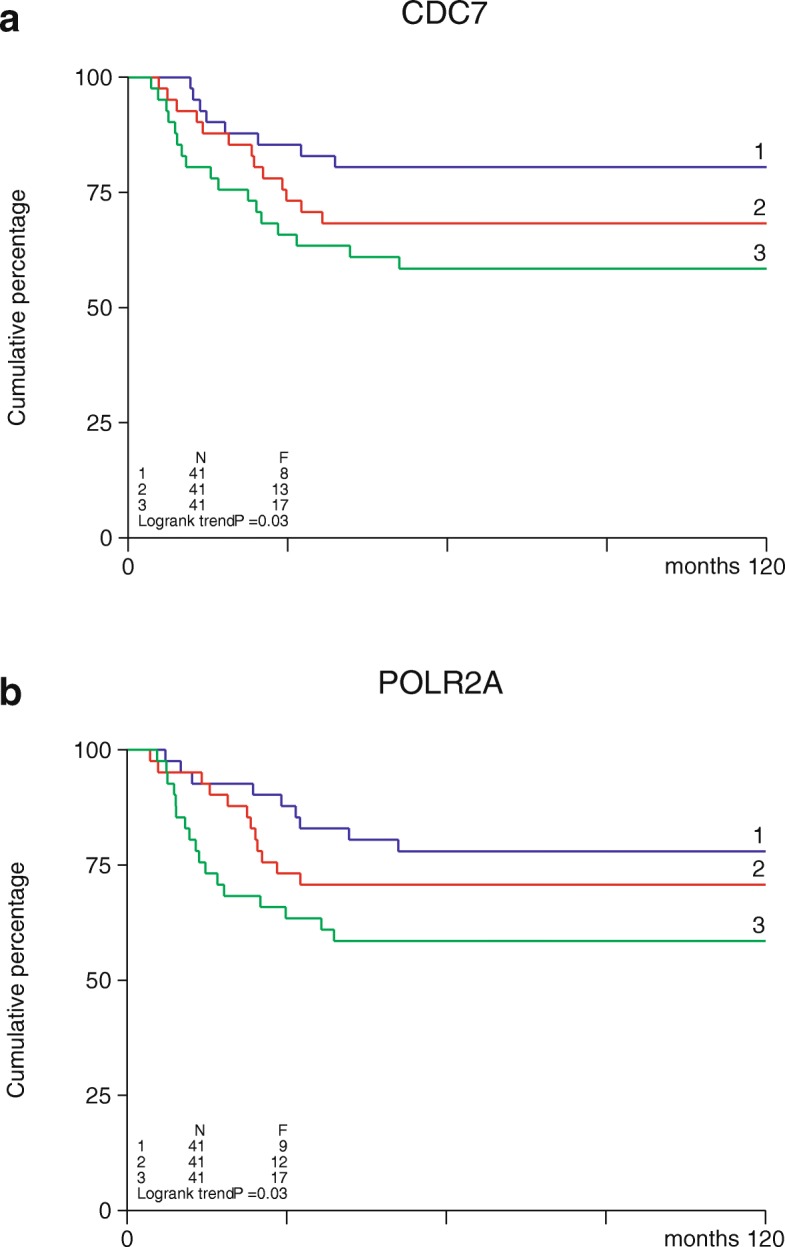
Expression of cdc7 and RNAII (POLR2A) is linked to poor prognosis in breast cancer patients. Metastasis-free survival curves illustrating the relationship between expression of cdc7 or RNAII (POLR2A) and prognosis in ER-negative breast cancer patients. Curves derived from gene expression and available survival data for 123 lymph node-negative, non-neoadjuvantly treated, ER-negative breast cancer patients at Erasmus MC Rotterdam. N = the number of patients in the group. F = the number of patients who relapsed (distant metastasis)

Next, in relation to an in-house cohort of lymph node-negative, non-(neo) adjuvantly treated 123 oestrogen receptor-negative (ER-neg) breast cancer patients, high expression of cdc7 was significantly associated with a poor metastasis-free survival (MFS) (Fig. 5a). Interestingly, high expression of POLR2A (RNA-II), the essential downstream target of CDK9, was also significantly associated with a poor metastasis-free survival in this patient group (Fig. 5b), though CDK9 expression level was not.

### Co-treatment of TNBC cells with lapatinib and PHA-767491 inhibits crucial signalling networks and the expression of genes linked to poor survival in TNBC

**Fig. 6:**
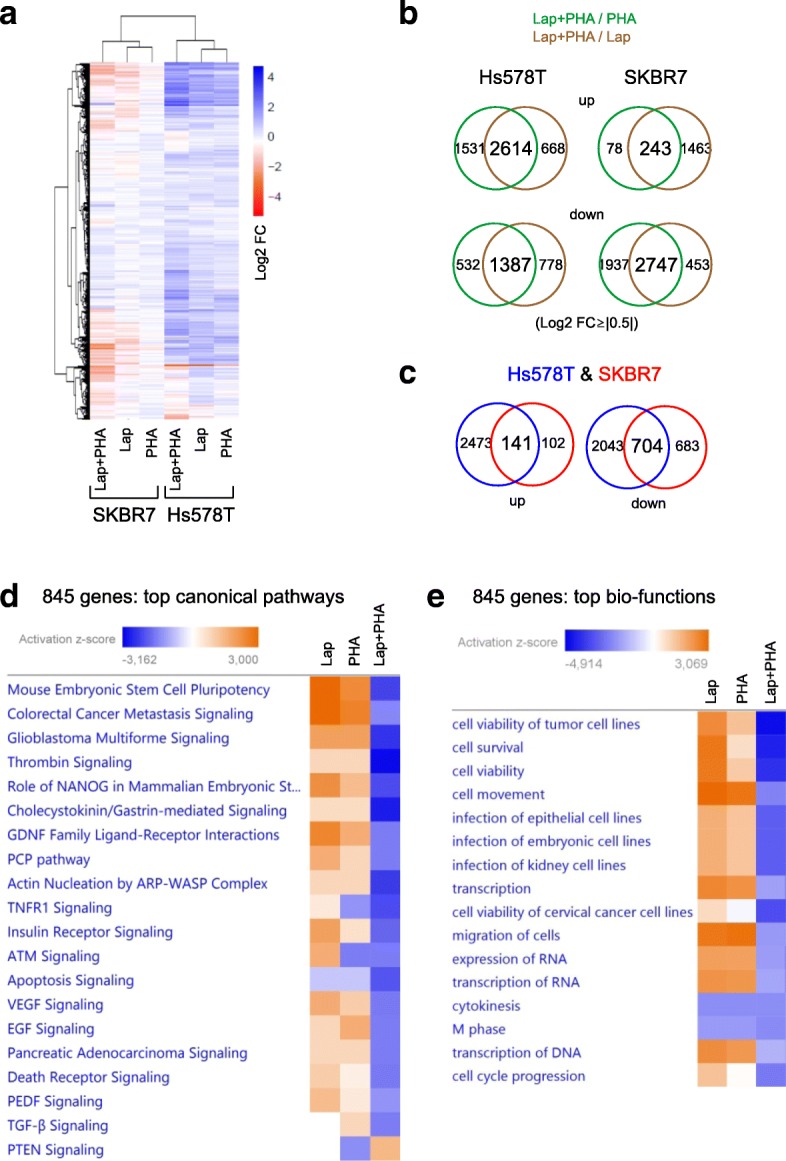
Co-treatment of TNBC cells with lapatinib and PHA-767491 inhibits crucial signalling networks in TNBC. **a** Global transcriptomic signature in SKBR7 and Hs578T cells after 6 h of treatment with lapatinib (3.16 μM), PHA-767491 (1 μM) or a combination of these two doses. Log_2_ fold change (FC) normalised to DMSO control shown. **b** Venn diagrams showing the number of genes significantly (Log_2_ FC ≥ 0.5) up- or downregulated after monotherapy and combination therapy in Hs578T and SKBR7 cells. **c** Venn diagrams showing the number of genes commonly and significantly (Log_2_ FC ≥ 0.5) up- or downregulated after combination therapy in both Hs578T and SKBR7 cells. **d** Top canonical pathways enriched in 845 significantly differentially expressed genes in combination therapy as predicted by IPA online analysis. *Z*-score indicates activation (orange) or inhibition (blue) of the pathway under indicated conditions, respectively. **e** Top bio-functions enriched in 845 significantly differentially expressed genes in combination therapy conditions as predicted by IPA software

**Fig. 7:**
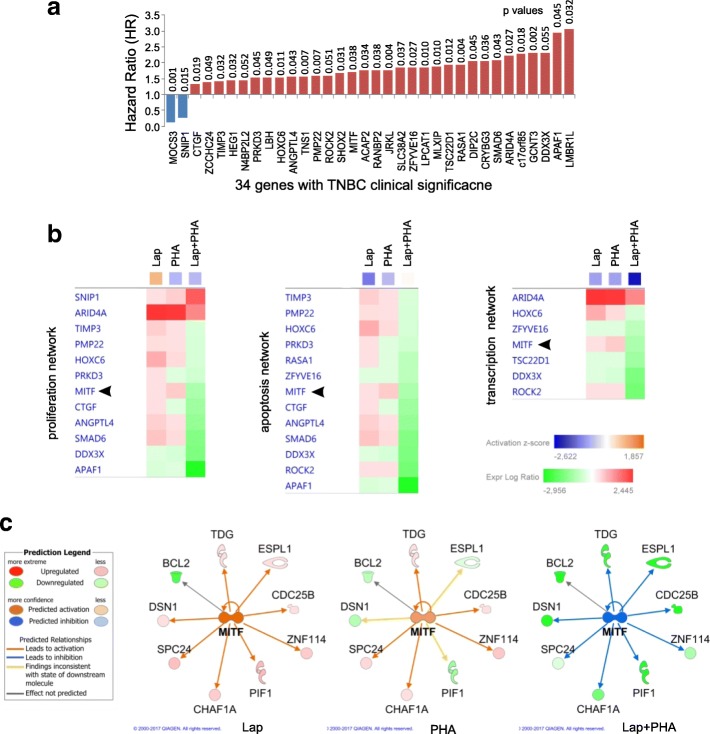
RNA-Seq reveals genes linked to poor survival in TNBC which are involved in the regulation of transcription, apoptosis and proliferation. **a** Thirty-four genes specifically downregulated by combination therapy whose expression is significantly correlated with metastasis-free survival in 142 lymph node-negative, non-neoadjuvantly treated, TNBC patients (data derived from in-house cohort as well as from publicly available datasets). HR hazard ratio. **b** Presence of aforementioned clinically relevant, downregulated genes in the top biological networks inhibited by combination therapy. **c** MITF, an upstream regulator of genes which are downregulated in response to inhibition of MITF under combination therapy

To investigate how combined EGFR-TKI and PHA-767491 treatment distorts the global transcriptional signature of TNBC cells, we performed RNA sequencing in Hs578T and SKBR7 cells treated with monotherapies or combination therapy (Fig. 6a). We identified 2614 genes upregulated by co-treatment in Hs578T cells, with 243 genes being upregulated in SKBR7 cells under the same conditions (Fig. 6b). 1387 and 2747 genes were downregulated in co-treated Hs578T and SKBR7 cells, respectively (Fig. 6b). Amongst these genes, 141 up- and 704 downregulated genes (845 transcripts) were commonly responsive to co-treatment in both Hs578T and SKBR7 cells (Fig. 6c). Integrated pathway analysis of these 845 synergy-related genes revealed striking disturbance of pro-proliferative pathways mediated by growth factors such as TGF-β, EGF and insulin, as well as pathways governing angiogenesis, stem cell pluripotency and metastatic signalling (Fig. 6d). More specifically, an enrichment of genes related to cell survival, viability and migration as well as RNA transcription, cytokinesis, mitosis and cell cycle progression was found amongst the genes commonly downregulated by co-treatment (Fig. 6e). Amongst the top biological networks downregulated as a result of combination therapy, cell survival, transcription and cell cycle progression were particularly prominent (Additional file 6: Figure S4). Thirty-four (2 up- and 32 downregulated) genes out of the 845 lapatinib and PHA-767491 co-targeted genes were significantly correlated with metastasis-free survival (MFS) in 142 lymph node-negative, non-neoadjuvantly treated TNBC patients, including LMBR1L, APAF1, DDX3X and GCNT3, with hazard ratios > 2 (Fig. 7a and Additional file 7: Table S3). These 34 clinically relevant genes were present in the main biological networks of apoptosis, transcription and proliferation (Fig. 7b), in which MITF, HOXC6 and ROCK2 were all involved. MITF was defined as a regulator known to influence the levels of anti-apoptotic protein BCL-2 as well as transcriptional regulators (ZNF114), G2/M regulators (CDC25B), mitotic regulators (DSN1) and DNA replication/repair proteins (TDG and PIF-1), these being downregulated in response to MITF inhibition (Fig. 7c).

## Discussion

EGFR is highly expressed in both TNBC tumours and cell lines, supporting a role for EGFR as an oncogenic driver in TNBC. However, clinical trials suggest single inhibition of EGFR signalling is incapable of eliminating TNBC cells [17, 18, 21, 31]. Consistently, our results demonstrated that targeting EGFR kinase activity by EGFR-TKIs, including lapatinib, erlotinib and gefitinib, insufficiently inhibits TNBC cell proliferation, despite inhibition of EGFR phosphorylation. Our kinase inhibitor combination screen demonstrated that the dual cdc7/CDK9 inhibitor PHA-767491 enables EGFR-TKIs to inhibit proliferation, induce G2-M cell cycle arrest and promote apoptosis in various TNBC cell lines expressing high levels of EGFR. This synergistic drug interaction downregulates the activity of components of the transcription apparatus and the DNA replication programme, including cdc7, CDK9, pMCM2 (S40/41), p-RNAII (S2/5), CDK4, cyclin D1 and Rb, making the combination of EGFR and cdc7/CDK9 molecular-targeted therapies promising for this subgroup of breast cancer.

CDK9 is a member of positive elongation factor P-TEFb and together with CDK7 is vital for gene transcription since CDK7 and CDK9 sequentially phosphorylate the C-terminal domain (CTD) of RNA Polymerase II (RNA II) at Ser5/Ser7 and Ser2, respectively, allowing dissociation of negative elongation factors and subsequent elongation of mRNA transcripts [32–34]. Blockage of this critical elongation step results in stalled transcription which triggers ubiquitination of RNAII at active gene promoters and its subsequent proteasome-mediated degradation [35]. Given that co-treatment with higher concentrations of PHA-767491 reduced total levels of RNAII, investigating whether CDK9 inhibition-mediated transcriptional stalling is responsible for proteasome-dependent depletion of this protein is prudent. Previous studies have demonstrated the potential of inhibiting CDK9 in in vitro and in vivo PDX models of TNBC using pan-CDK inhibitor dinaciclib, leading to G2/M cell cycle arrest and apoptosis, consistent with the results presented herein [36]. CDK9 is essential for the growth of both HR+ and TNBC cell lines, whilst EGFR is one of many “Achilles’cluster” genes sensitive to CDK7 inhibition and vital for TNBC survival [37]. Consistently, we showed that despite being resistant to inhibition of EGFR kinase activity by various EGFR-TKIs, complete silencing of EGFR is detrimental to TNBC cell growth. In addition, EGFR is capable of acting as a transcription factor [38, 39]. The nuclear translocation of EGFR is associated with resistance to chemotherapeutics in TNBC [38, 40–42] and shields the RTK from the effects of TKIs limited to the cell membrane, permitting EGFR to enhance transcription of genes which govern cell cycle progression, such as Cyclin D1 and Aurora Kinase [39, 43]. Cdc7 kinase is itself indispensable for correct regulation of cell cycle progression, exerting control over both initiation of DNA replication and the DNA damage response [44, 45]. By phosphorylating mini-chromosome maintenance proteins (MCM2-7) present in pre-replicative complexes formed during G1 phase, cdc7 activates the helicase activity of these proteins, leading to unwinding of DNA strands and thereby initiating DNA replication at the G1-S phase checkpoint [46, 47]. It also has been reported that EGFR indirectly influences the initiation of DNA replication by eliciting phosphorylation of MCM7 in a Lyn kinase-dependent fashion, thereby delineating possible functional overlap between cdc7 and EGFR [48]. TNBC cells often possess p53-inactivating mutations which abolish the DNA replication origin activation checkpoint, rendering them susceptible to the induction of replicative stress [49]. Induction of G2-M arrest in our TNBC cell lines after combined inhibition of EGFR and cdc7/CDK9 is consistent with data from other studies which demonstrated that a p53-dependent checkpoint is critical for mitigating aberrant cell cycle progression after cdc7 depletion [50, 51].

The CDK4/Cyclin D1 complex phosphorylates Rb at Ser780/795 thereby inactivating Rb in G1/S checkpoint regulation [52, 53]. Consistently, co-inhibition of cdc7/CDK9 and EGFR signalling in our TNBC cell lines reduces CDK4 and Cyclin D1 levels accompanied by reduced phosphorylation of Rb, thereby resulting in G2-M arrest and ultimately apoptosis. Whether the downregulation of Cyclin D1 and CDK4 by EGFR-TKIs and PHA-767491 represents a global decrease in the transcription of rapidly expressed, immediate response genes due to inhibition of CDK9-mediated transcriptional elongation, a decrease in the transcriptional activity of EGFR, or a by-product of the cell cycle arrest induced by cdc7 depletion, merits further investigation. Taken together, these results identify possible functional links between signalling downstream of EGFR and the function of both cdc7 and CDK9, which may to some extent explain the observed synergy between EGFR-TKIs and PHA-767491. Additionally, silencing of cell cycle-regulatory or transcriptional CDKs in combination with a cdc7-specific inhibitor (XL413) in breast cancer cells has been shown to mimic the cell cycle disruption caused by PHA-767491 [54]. Silencing of CDK9 led to negligible impact on progression of MCF10A cells through S-phase, whilst CDK9-depleted cells treated with XL413 accumulated in late S-phase, suggesting that the profound cell cycle arrest in TNBC cells caused by PHA-767491 or cdc7 depletion may be somewhat dependent on CDK9 or can at least be augmented by inhibiting CDK9. Nonetheless, using RNAi-mediated silencing of cdc7 and CDK9, we were unable to fully recapitulate the observed synergy between EGFR-TKIs and PHA-767491 in selected TNBC cell lines. Although PHA-767491 has off-target effects on CDK1, CDK2 and GSK-3β which could contribute to sensitisation of TNBC cells to EGFR-TKIs, also knockdown of these genes had limited effects on the response to lapatinib. Despite that triple combination of the two other selective cdc7 and CDK9 inhibitors together with lapatinib strongly affected proliferation in the TNBC cell lines, this effect was not as pronounced as the combination of lapatinib with PHA-767491. This suggests that besides cdc7/CDK9 blockage by PHA-767491, also inhibition of other kinases likely contributes to the observed synergy. Broad spectrum kinase inhibition is not uncommon for highly effective anticancer therapeutics used in the clinic. Here we have only tested the PHA-767491/Lap combination in TNBC cell lines. Given the broader anti-kinase activity of PHA-767491 and the side effects of lapatinib and other EGFR inhibitors on the liver and/or heart, further assessment of the safety of such a combination treatment will be essential.

RNA-Seq transcriptomics identified genes specifically downregulated by co-treatment with EGFR-TKIs and PHA-767491, which were involved in pathways regulating survival, transcription and cell cycle progression. The decreased expression of these genes (a number of them associated with poorer MFS in TNBC) by PHA-767491 combined with inhibition of EGFR leads to apoptosis and downregulation of transcription and proliferation. Interestingly, the transcription factor MITF (microphthalamia-associated transcription factor), a major upstream regulator of pathways governing apoptosis, proliferation and transcription, was decreased together with its downstream targets pro-survival BCL-2 and cell cycle-regulatory CDC25B as a result of combining lapatinib and PHA-767491. With regards to TNBC, little is known about MITF’s contribution to EGFRi-resistant phenotypes. Further research is therefore required to validate whether targeting of MITF function constitutes a logical therapeutic avenue in TNBC, or whether MITF inhibition is sufficient to reverse the resistance of TNBC cells to EGFR-targeted therapies.

## Conclusions

In summary, we show that multiple EGFR-TKIs synergise with the dual cdc7/CDK9 inhibitor PHA-767491 in various EGFR-TKI-resistant TNBC cell lines resulting in decreased proliferation, induction of apoptosis and G2/M cell cycle arrest. Combination therapy leads to inhibition of proteins crucial for accurate DNA replication and CDK9/RNAII-mediated gene transcription. This combination also leads to inhibition of crucial pro-oncogenic networks and reduces the expression of genes linked to ERK/mTOR signalling and poor progression-free survival in TNBC patients, perhaps identifying possible candidate genes for further research into the mechanism of this synergy and as therapeutic targets.

## Additional files

Additional file 1: **Table S1.** Cell seeding densities. Densities used per TNBC cell line for the kinase inhibitor library screening. Densities are shown as number of cells/well in 96-well plates. (XLSX 10 kb)

Additional file 2: **Table S2.** Composition of in-house ER-negative breast cancer patient cohort. (XLSX 21 kb)

Additional file 3: **Figure S1.** Resistance of TNBC cells to EGFR-TKIs. a. Effect of lapatinib on induction of apoptosis in selected lapatinib-resistant and lapatinib-sensitive TNBC cell lines. Hs578T, BT549, SKBR7 and HCC1806 cells were treated with lapatinib (3.16 μM) as indicated, for 24 h, 48 h or 72 h, respectively, stained with Annexin-V and Hoechst, followed by imaging and image quantification. Relative cell death was quantified by normalising the intensity of Annexin-V signal to that of DMSO control. One-way ANOVA **** *P* ≤ 0.0001, *** *P* ≤ 0.001, ** *P* ≤ 0.01, * *P* ≤ 0.05. b. Impact of silencing EGFR and downstream components of EGFR signalling pathway on the proliferation of Hs578T cells. Hs578T cells were transfected with siRNAs targeting EGFR, ERK2 and FRAP1 as well as positive (siKIF11) and negative controls (siGAPDH and siKinase Pool) as described and grown for 4 days. Proliferation was then assessed using sulphorhodamine B assay. Results were normalised to Kinase Pool using the % control method as described in materials and methods. (PDF 191 kb)

Additional file 4: **Figure S2.** EGFR-negative TNBC cell line SUM185PE is insensitive to co-treatment with lapatinib and PHA-767491. a. Immunofluorescence imaging of EGFR-positive (SKBR7) and EGFR-negative (SUM185PE) TNBC cell lines. Cells were fixed using 1% paraformaldehyde and 0.1% Triton-X for 15 min before being washed thrice with 1x PBS and blocked with 0.5% bovine serum albumin in PBS for 30 min. EGFR antibody (sc-03; Santa Cruz Biotechnology®) was used to stain EGFR overnight at 4 °C. Fixed cells were then incubated with anti-rabbit Alexa 488-conjugated secondary antibody (A11008; Molecular Probes®) or Hoechst (nuclear stain; 1:10,000) for 1 h at room temperature in the dark before being imaged at 20x magnification. b. Impact of EGF stimulation on EGFR-mediated signal transduction in EGFR-negative cell line SUM185PE. Cells were starved overnight in serum-free medium before being treated with lapatinib (3.16 μM) for 4 h and subsequently stimulated with EGF (100 ng/ml) for 5 min. Cells were then lysed and protein samples subjected to SDS-PAGE and immunoblotting with indicated antibodies. **c.** Dose-response experiment combining lapatinib and PHA-767491 in SUM185PE cells. The upper graph shows the response of SUM185PE cells to lapatinib and PHA-767491 monotherapies. The lower graph displays the proliferation of SUM185PE cells after combining lapatinib (3.16 μM) with dose range (0.01–10 μM) of PHA-767491. Proliferation was assessed using sulphorhodamine B assay and results were normalised to DMSO using the % control method as outlined in materials and methods. (PDF 525 kb)

Additional file 5: **Figure S3.** EGFR-TKIs and PHA-767491 synergise to induce G2/M cell cycle arrest and apoptosis. a. Cell cycle distribution histograms of Hs578T and SKBR7 cells after 48-h treatment with EGFR-TKIs (lapatinib, erlotinib or gefitinib at 3.16 μM) alone or combined with PHA-767491 (1 μM or 3.16 μM), as indicated. b. Induction of apoptosis by EGFR-TKI and PHA-767491 co-treatment. SKBR7 and SUM149PT cells were treated with lapatinib (3.16 μM), PHA-767491 (3.16 μM), alone or combined, as indicated, for 24 h, 48 h or 72 h, respectively, and then stained with Annexin-V and Hoechst, followed by imaging and image quantification. Relative cell death was quantified by normalising the intensity of Annexin-V signal to that of DMSO control. One-way ANOVA ****P ≤ 0.0001, ***P ≤ 0.001, **P ≤ 0.01, *P ≤ 0.05. (PDF 378 kb)

Additional file 6: **Figure S4.** siRNA-mediated silencing of cdc7 and/or CDK9 in combination with lapatinib. a. Silencing cdc7 or CDK9 synergises with lapatinib to inhibit proliferation in SKBR7 cells but not Hs578T or BT549 cells. % proliferation 4 days post-treatment with lapatinib (3.16 μM) or DMSO (1:1000) was normalised to Kinase Pool using % control method outlined in materials and methods. One-way ANOVA **P ≤ 0.01, *P ≤ 0.05, ns: not significant b. Effect of siCdc7 and siCDK9 double knockdown in Hs578T cells. c. Effect of siCdc7 and siCDK9 on cdc7 and CDK9-mediated signal transduction in Hs578T, BT549 and SKBR7 cells. Cells were transfected with control (siKinase Pool (−)), siCDK9 or siCdc7 then treated with lapatinib (3.16 μM) or DMSO, and lysates were extracted 48 h post-exposure to compounds and subjected to immunoblotting. d. Anti-proliferative effects of knockdown of off-targets of PHA-767491 in combination with lapatinib (3.16 μM) or DMSO. e. Effects of selective inhibition of cdc7 (TAK-931) and CDK9 (BAY-1143572) in combination with lapatinib on proliferation of Hs578T and SKBR7 cells. (PDF 1727 kb)

Additional file 7: **Table S3.** 34 clinically relevant genes specifically downregulated after co-treatment with lapatinib and PHA-767491 in Hs578T and SKBR7 cells. (XLSX 10 kb)

## Data Availability

RNA-Seq data generated in this study have been submitted to the NCBI Gene Expression Omnibus (GEO) under accession number GSE131375. The cell line transcriptomics and TNBC patient gene expression datasets analysed in this study were available from the GEO data repository (GSE41313) and the TCGA-BRCA database respectively. All other data are included in this article and its supplementary files.

## Abbreviations

AKT: v-akt murine thymoma viral oncogene homologue
BL1: Basal-like 1 subtype
BL2: Basal-like 2 subtype
Cdc7: Cell division cycle 7-related protein kinase
CDK4: Cyclin-dependent kinase 4
CDK7: Cyclin-dependent kinase 7
CDK9: Cyclin-dependent kinase 9
CI: Combination index
CTD: C-terminal domain
EGFR: Epidermal growth factor receptor
EGFR-TKI: Epdiermal growth factor receptor tyrosine kinase inhibitor
ERK1/2: Extracellular signal-regulated kinase 1/2
HER2: Human epidermal growth factor receptor 2
HR+/−: Hormone receptor-positive/negative
IM: Immunomodulatory subtype
LAR: Luminal androgen receptor-positive subtype
M: Mesenchymal subtype
MCM2: Mini-chromosome maintenance protein 2
MEK: Mitogen-activated protein kinase kinase
MITF: Microphthalamia-associated transcription factor
MSL: Mesenchymal stem-like subtype
POLR2A: RNA Polymerase II subunit 2A
P-TEFb: Positive transcription elongation factor b
Rb: Retinoblastoma protein
RNA II: RNA Polymerase II
S2/5: Serine 2/5 of RNA polymerase II subunit 2A’s C-terminal domain
SRB: Sulphorhodamine B
TNBC: Triple-negative breast cancer
