# Supplementary figures and images for: A kinase inhibitor screen identifies a dual cdc7/CDK9 inhibitor to sensitise triple-negative breast cancer to EGFR-targeted therapy
Source: Breast Cancer Res. 2019 Jul 1;21:77. doi: 10.1186/s13058-019-1161-9 (PMC6604188; doi:10.1186/s13058-019-1161-9)

**a**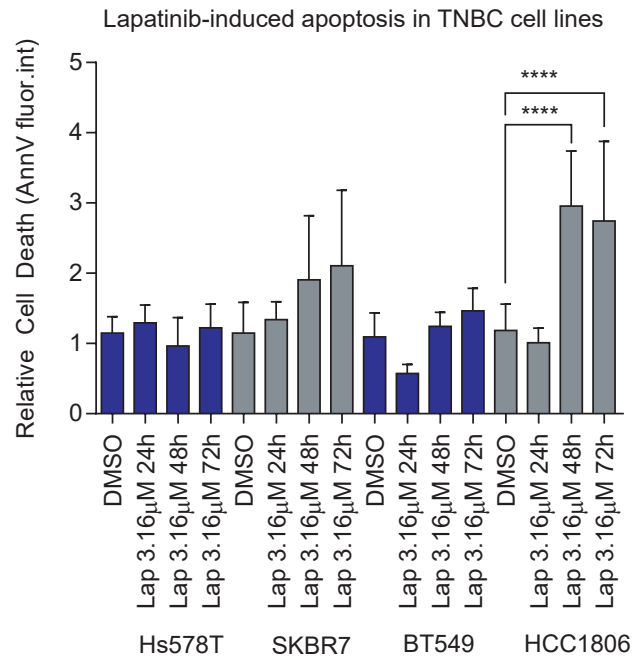**b**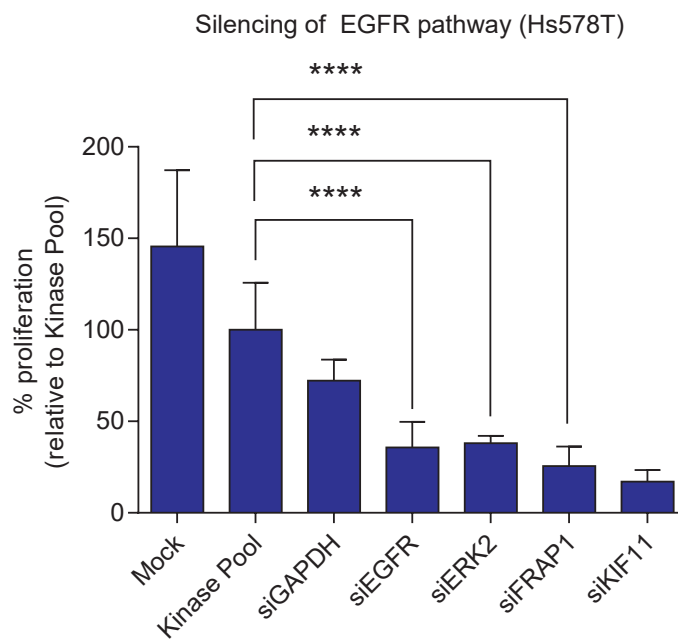

Supplement: Supplementary file 3 — Figure S1. Resistance of TNBC cells to EGFR-TKIs. a. Effect of lapatinib on induction of apoptosis in selected lapatinib-resistant and lapatinib-sensitive TNBC cell lines. Hs578T, BT549, SKBR7 and HCC1806 cells were treated with lapatinib (3.16 μM) as indicated, for 24 h, 48 h or 72 h, respectively, stained with Annexin-V and Hoechst, followed by imaging and image quantification. Relative cell death was quantified by normalising the intensity of Annexin-V signal to that of DMSO control. One-way ANOVA **** P ≤ 0.0001, *** P ≤ 0.001, ** P ≤ 0.01, * P ≤ 0.05. b. Impact of silencing EGFR and downstream components of EGFR signalling pathway on the proliferation of Hs578T cells. Hs578T cells were transfected with siRNAs targeting EGFR, ERK2 and FRAP1 as well as positive (siKIF11) and negative controls (siGAPDH and siKinase Pool) as described and grown for 4 days. Proliferation was then assessed using sulphorhodamine B assay. Results were normalised to Kinase Pool using the % control method as described in materials and methods. (PDF 191 kb) [file 13058_2019_1161_MOESM3_ESM.pdf]

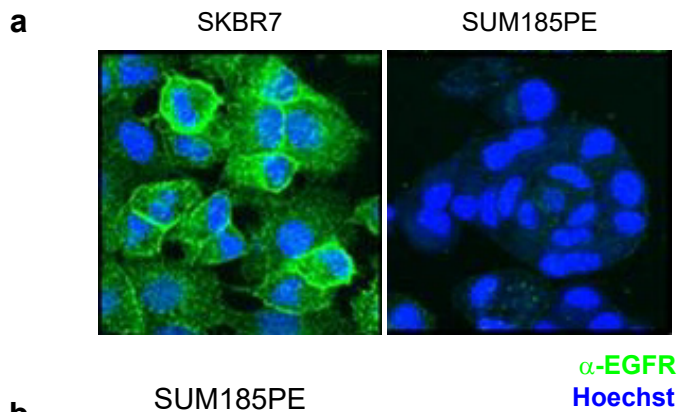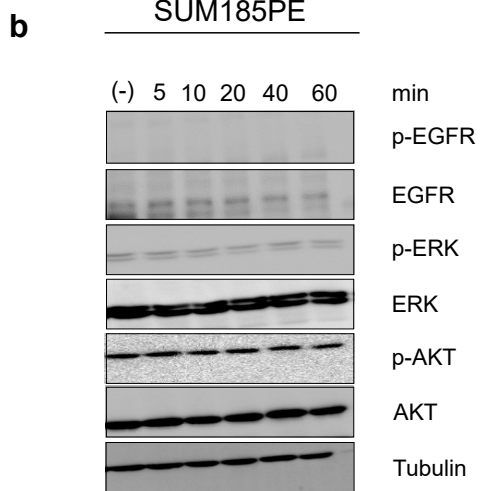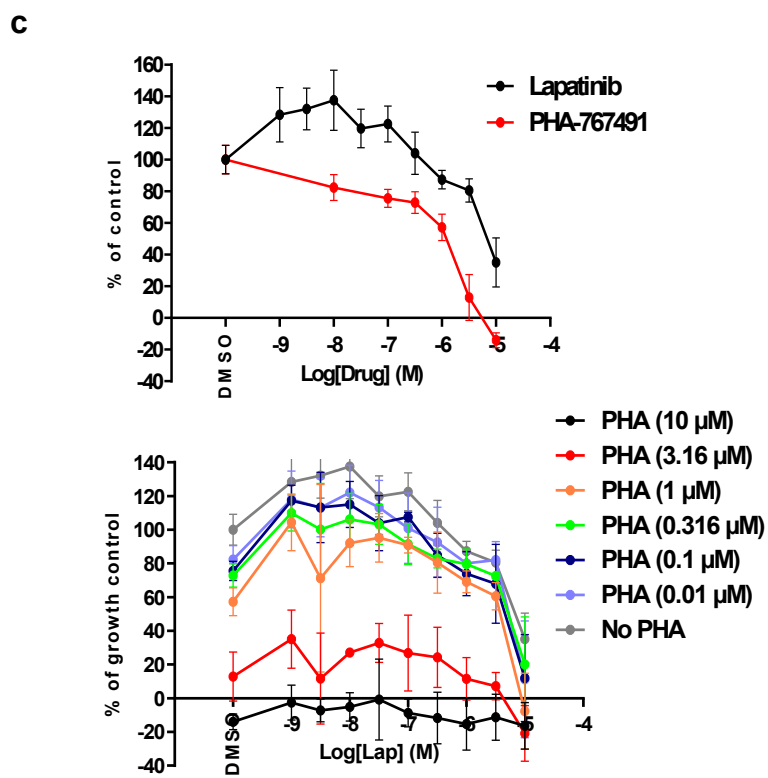

Supplement: Supplementary file 4 — Figure S2. EGFR-negative TNBC cell line SUM185PE is insensitive to co-treatment with lapatinib and PHA-767491. a. Immunofluorescence imaging of EGFR-positive (SKBR7) and EGFR-negative (SUM185PE) TNBC cell lines. Cells were fixed using 1% paraformaldehyde and 0.1% Triton-X for 15 min before being washed thrice with 1x PBS and blocked with 0.5% bovine serum albumin in PBS for 30 min. EGFR antibody (sc-03; Santa Cruz Biotechnology®) was used to stain EGFR overnight at 4 °C. Fixed cells were then incubated with anti-rabbit Alexa 488-conjugated secondary antibody (A11008; Molecular Probes®) or Hoechst (nuclear stain; 1:10,000) for 1 h at room temperature in the dark before being imaged at 20x magnification. b. Impact of EGF stimulation on EGFR-mediated signal transduction in EGFR-negative cell line SUM185PE. Cells were starved overnight in serum-free medium before being treated with lapatinib (3.16 μM) for 4 h and subsequently stimulated with EGF (100 ng/ml) for 5 min. Cells were then lysed and protein samples subjected to SDS-PAGE and immunoblotting with indicated antibodies. c. Dose-response experiment combining lapatinib and PHA-767491 in SUM185PE cells. The upper graph shows the response of SUM185PE cells to lapatinib and PHA-767491 monotherapies. The lower graph displays the proliferation of SUM185PE cells after combining lapatinib (3.16 μM) with dose range (0.01–10 μM) of PHA-767491. Proliferation was assessed using sulphorhodamine B assay and results were normalised to DMSO using the % control method as outlined in materials and methods. (PDF 525 kb) [file 13058_2019_1161_MOESM4_ESM.pdf]

**a**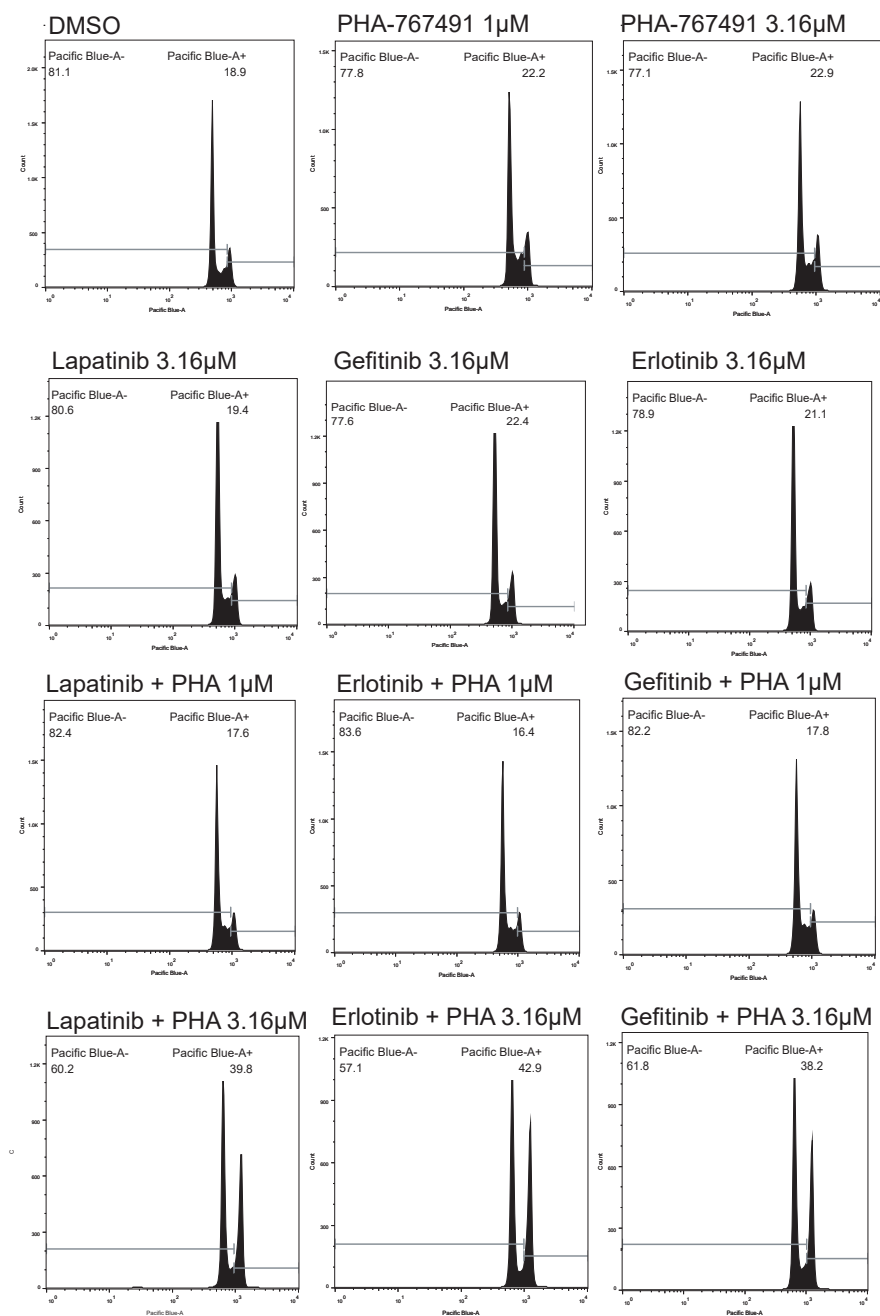**b**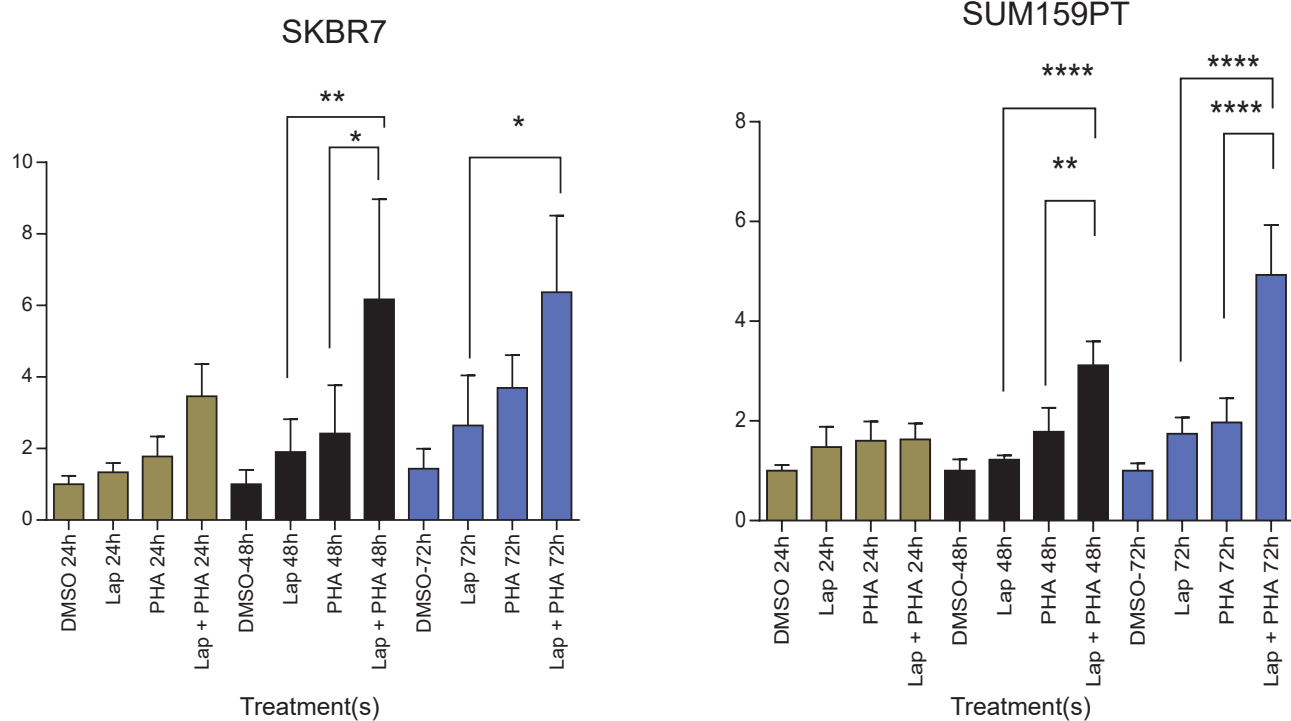

Supplement: Supplementary file 5 — Figure S3. EGFR-TKIs and PHA-767491 synergise to induce G2/M cell cycle arrest and apoptosis. a. Cell cycle distribution histograms of Hs578T and SKBR7 cells after 48-h treatment with EGFR-TKIs (lapatinib, erlotinib or gefitinib at 3.16 μM) alone or combined with PHA-767491 (1 μM or 3.16 μM), as indicated. b. Induction of apoptosis by EGFR-TKI and PHA-767491 co-treatment. SKBR7 and SUM149PT cells were treated with lapatinib (3.16 μM), PHA-767491 (3.16 μM), alone or combined, as indicated, for 24 h, 48 h or 72 h, respectively, and then stained with Annexin-V and Hoechst, followed by imaging and image quantification. Relative cell death was quantified by normalising the intensity of Annexin-V signal to that of DMSO control. One-way ANOVA ****P ≤ 0.0001, ***P ≤ 0.001, **P ≤ 0.01, *P ≤ 0.05. (PDF 378 kb) [file 13058_2019_1161_MOESM5_ESM.pdf]
