# Supplementary material for: A kinase inhibitor screen identifies a dual cdc7/CDK9 inhibitor to sensitise triple-negative breast cancer to EGFR-targeted therapy
Source: Breast Cancer Res. 2019 Jul 1;21:77. doi: 10.1186/s13058-019-1161-9 (PMC6604188; doi:10.1186/s13058-019-1161-9)

a

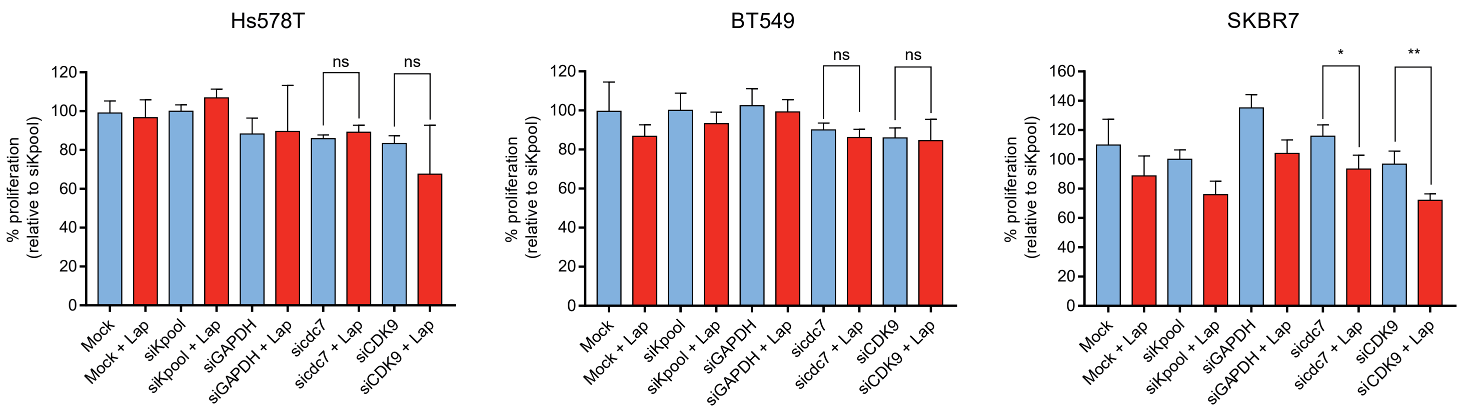

b

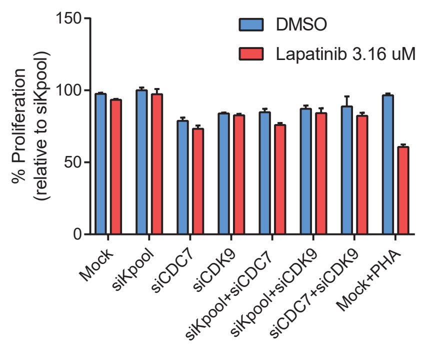

c

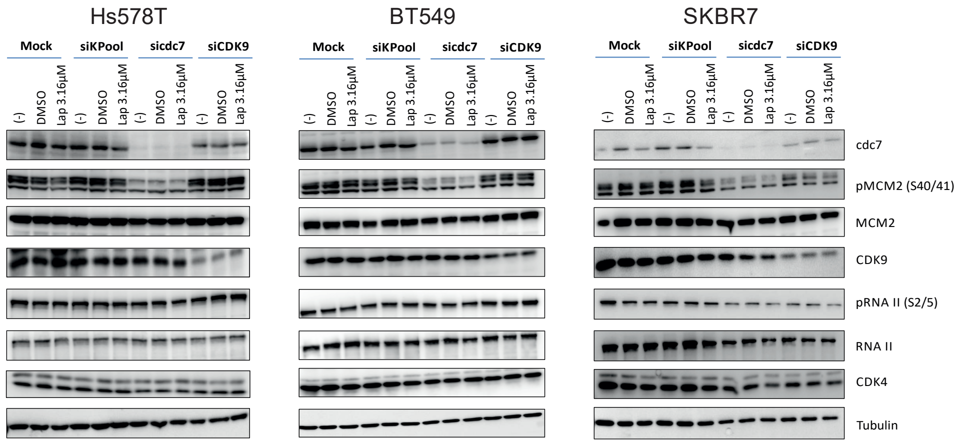

d

**PHA-767491 targets**	**Kinase inhibition (IC50)**
CDC7	10 nM
CDK9	34 nM
GSK3b	220 nM
CDK2	240 nM
CDK1 (CDC2)	250 nM
CDK5	460 nM
MK2 (MAPKAPK2)	470 nM
PLK1	980 nM

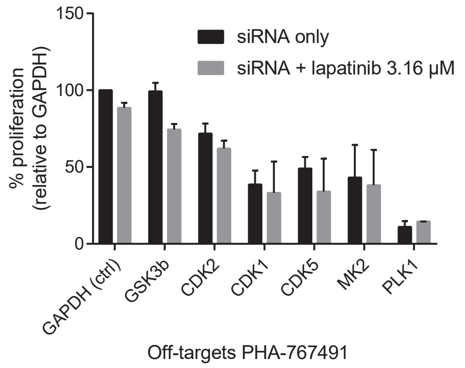

e

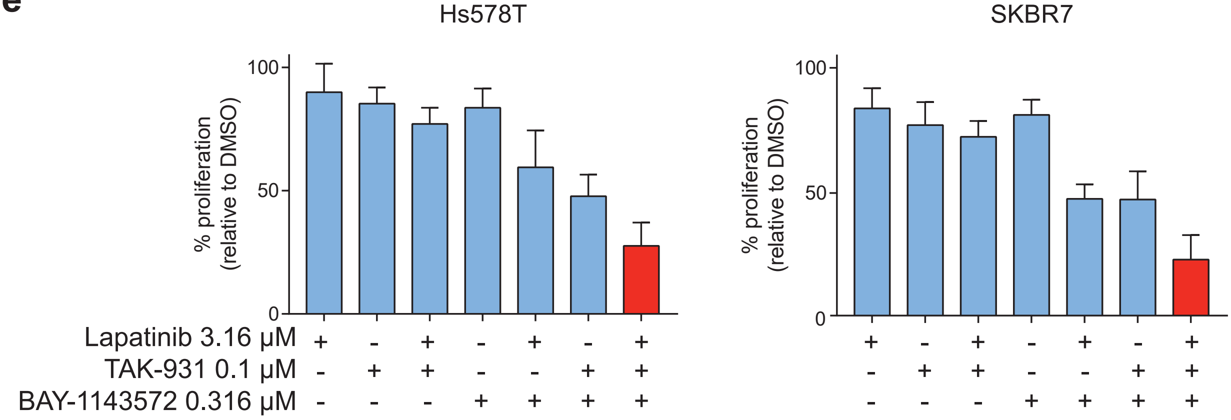

Supplement: Supplementary file 6 — Figure S4. siRNA-mediated silencing of cdc7 and/or CDK9 in combination with lapatinib. a. Silencing cdc7 or CDK9 synergises with lapatinib to inhibit proliferation in SKBR7 cells but not Hs578T or BT549 cells. % proliferation 4 days post-treatment with lapatinib (3.16 μM) or DMSO (1:1000) was normalised to Kinase Pool using % control method outlined in materials and methods. One-way ANOVA **P ≤ 0.01, *P ≤ 0.05, ns: not significant b. Effect of siCdc7 and siCDK9 double knockdown in Hs578T cells. c. Effect of siCdc7 and siCDK9 on cdc7 and CDK9-mediated signal transduction in Hs578T, BT549 and SKBR7 cells. Cells were transfected with control (siKinase Pool (−)), siCDK9 or siCdc7 then treated with lapatinib (3.16 μM) or DMSO, and lysates were extracted 48 h post-exposure to compounds and subjected to immunoblotting. d. Anti-proliferative effects of knockdown of off-targets of PHA-767491 in combination with lapatinib (3.16 μM) or DMSO. e. Effects of selective inhibition of cdc7 (TAK-931) and CDK9 (BAY-1143572) in combination with lapatinib on proliferation of Hs578T and SKBR7 cells. (PDF 1727 kb) [file 13058_2019_1161_MOESM6_ESM.pdf]
